# 2319. Trends in Health Services Utilization among Veterans with HIV Before and During the COVID-19 Pandemic

**DOI:** 10.1093/ofid/ofad500.1941

**Published:** 2023-11-27

**Authors:** Ruth O Adekunle, Ahmed Mohamed, Charlene Pope, Mulugeta Gebregziabher, Robert Axon

**Affiliations:** Medical University of South Carolina, Charleston, South Carolina; Ralph H. Johnson VA Medical Center, Charleston, South Carolina; Ralph H. Johnson VA Medical Center, Charleston, South Carolina; Ralph H. Johnson VA Medical Center, Charleston, South Carolina; Ralph H. Johnson VA Medical Center, Charleston, South Carolina

## Abstract

**Background:**

Public health efforts to curb the spread of COVID-19 required structural changes to healthcare delivery, necessitating the decrease of in-person visits and the transition to virtual visits. Without sustained adherence to antiretroviral therapy, Persons Living with HIV/AIDS (PLWH) experience increased morbidity and mortality. It is unknown how disruptions in access to healthcare as a result of the COVID-19 pandemic impacted care retention of Veterans with HIV (VWH). We aimed to compare visit volumes for HIV care before and during the ongoing COVID-19 pandemic.

**Methods:**

VWH who receive their care in the Veteran Affairs Health Care System between January 1, 2019, and December 31, 2021, were included in the study. Outpatient visits and inpatient stays for all cohort members were extracted from the Corporate Data Warehouse. Interrupted time series analysis (ITSA) was used to test for healthcare interruption around the date of COVID-19 restriction implementation (end of March 2020 or on 2020 week 15). An additional interruption point was added on week 15 of 2021 based on the data distribution (around the time vaccination became available).

**Results:**

Of the VWH, 34,531 had at least one inpatient or outpatient visit during the study period. The number of distinct patients was approximately similar through 2019 (N=32,391), 2020 (N=32,002), and 2021 (N=31,924). However, the number of total inpatient stays and outpatient visits decreased from 826,317 in 2019 to 728,206 in 2020. ITSA showed a statistically significant reduction (-1790.247; 95% CL [-2724, -854]) in the total number of outpatient visits and inpatient stays around the implementation of COVID-19 restriction around the end of March 2020 followed by an increasing trend (slope= 142, 95% CL [115, 170]) (Figure 1). The second interruption point (intercept= -1250; 95 CL [-2308, -191]) was at 2021 week 15 demonstrating a negative trend [slope =-149, 96; 95% CL [-198, -99]).

Interrupted time series analysis of inpatient stays and outpatient visits for Veterans with HIV prior to and during the ongoing COVID-19 pandemic

**Figure:**
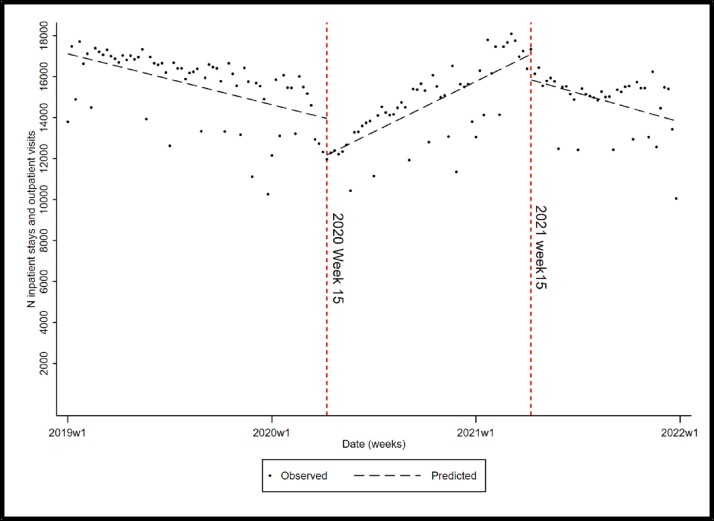

**Conclusion:**

Health services for VWH decreased during the peak of the pandemic, though utilization trends demonstrated that the number of visits was decreasing even before the COVID-19 pandemic. Despite the loosening of restrictions, healthcare utilization has not fully rebounded to pre-pandemic rates.

**Disclosures:**

**All Authors**: No reported disclosures

